# Probiotics [LGG-BB12 or RC14-GR1] versus placebo as prophylaxis for urinary tract infection in persons with spinal cord injury [ProSCIUTTU]: a study protocol for a randomised controlled trial

**DOI:** 10.1186/s12894-016-0136-8

**Published:** 2016-04-16

**Authors:** Bonsan Bonne Lee, Swee-Ling Toh, Suzanne Ryan, Judy M. Simpson, Kate Clezy, Laetitia Bossa, Scott A. Rice, Obaydullah Marial, Gerard Weber, Jasbeer Kaur, Claire Boswell-Ruys, Stephen Goodall, James Middleton, Mark Tudehope, George Kotsiou

**Affiliations:** Neuroscience Research Australia [NeuRA] and the University of New South Wales, Sydney, Australia; Department of Spinal and Rehabilitation Medicine, Prince of Wales Hospital, Sydney, Australia; School of Public Health, University of Sydney, Sydney, Australia; Department of Infectious Diseases, Prince of Wales Hospital, Sydney, Australia; Centre for Marine Bio-Innovation, University of New South Wales, Sydney, Australia; Royal Rehabilitation Centre Sydney, Sydney, Australia; Royal North Shore Hospital, Sydney, Australia; Centre for Health Economics Research and Evaluation [CHERE], University of Technology Sydney, Sydney, Australia; The Singapore Centre for Life Sciences Engineering and the School of Biological Sciences, Nanyang Technological University, Singapore, Singapore; John Walsh Centre for Rehabilitation Research, Kolling Institute, Northern Sydney Local Health District, St Leonards, NSW 2065 Australia; Sydney Medical School Northern, University of Sydney, Sydney, Australia

**Keywords:** Urinary prophylaxis, Multi-resistant organisms, Antibiotic resistance, Probiotics, Biofilm, Microbial community profiles

## Abstract

**Background:**

Urinary tract infections [UTIs] are very common in people with Spinal Cord Injury [SCI]. UTIs are increasingly difficult and expensive to treat as the organisms that cause them become more antibiotic resistant. Among the SCI population, there is a high rate of multi-resistant organism [MRO] colonisation. Non-antibiotic prevention strategies are needed to prevent UTI without increasing resistance. Probiotics have been reported to be beneficial in preventing UTIs in post-menopausal women in several *in vivo* and *in vitro* studies. The main aim of this study is to determine whether probiotic therapy with combinations of Lactobacillus reuteri RC-14 + Lactobacillus rhamnosus GR-1 [RC14-GR1] and/or Lactobacillus rhamnosus GG + Bifidobacterium BB-12 [LGG-BB12] are effective in preventing UTI in people with SCI compared to placebo.

**Method:**

This is a multi-site randomised double-blind double-dummy placebo-controlled factorial design study conducted in New South Wales, Australia. All participants have a neurogenic bladder as a result of spinal injury. Recruitment started in April 2011.

Participants are randomised to one of four arms, designed for factorial analysis of LGG-BB12 and/or RC14-GR1 v Placebo. This involves 24 weeks of daily oral treatment with RC14-GR1 + LGG-BB12, RC14-GR1 + placebo, LGG-BB12 + placebo or two placebo capsules. Randomisation is stratified by bladder management type and inpatient status. Participants are assessed at baseline, three months and six months for Short Form Health Survey [SF-36], microbiological swabs of rectum, nose and groin; urine culture and urinary catheters for subjects with indwelling catheters. A bowel questionnaire is administered at baseline and three months to assess effect of probiotics on bowel function.

The primary outcome is time from randomisation to occurrence of symptomatic UTI. The secondary outcomes are change of MRO status and bowel function, quality of life and cost-effectiveness of probiotics in persons with SCI. The primary outcome will be analysed using survival analysis of factorial groups, with Cox regression modelling to test the effect of each treatment while allowing for the other, assuming no interaction effect. Hazard ratios and Kaplan-Meier survival curves will be used to summarise results.

**Discussion:**

If these probiotics are shown to be effective in preventing UTI and MRO colonisation, they would be a very attractive alternative for UTI prophylaxis and for combating the increasing rate of antibiotic resistance after SCI.

**Trial registration:**

Australian New Zealand Clinical Trials Registry [ACTRN 12610000512022]. Date of registration: 21 June 2010.

**Electronic supplementary material:**

The online version of this article (doi:10.1186/s12894-016-0136-8) contains supplementary material, which is available to authorized users.

## Background

Urinary tract infections [UTIs] are very common in people with a neurogenic bladder. People with a spinal cord injury [SCI] and people with the spinal demyelinating form of Multiple Sclerosis [MS] are highly susceptible to the development of neurogenic bladder dysfunction.

UTIs have a high societal cost and current prevention strategies do not work. People with neurogenic bladder are at significant risk from UTI with approximately two [2] UTI episodes per year on average for persons with SCI [1]. One of the major clinical challenges for SCI patients and clinicians is that when patients get a UTI, simple oral antibiotics frequently are ineffective due to the high numbers of multi-resistant-organism [s] [MROs] within SCI populations [about 40–50 % of SCI people] [1, 2]. This greatly amplifies the health, societal and economic consequences of disease and can even lead to life threatening clinical problems that can spread if not controlled through hospitals and the community. Health care costs associated with cross infection are estimated at US$18–30 billion yearly in the USA and UK combined. Australian costs are expected to be proportionate [3]. Furthermore, based on the existing SCI UTI prevention literature, we have demonstrated that current commonly used methods of non-antibiotic UTI prevention in SCI do not work [4]. The prevention of UTI, particularly the more difficult to treat MRO UTI, is therefore a clinical imperative for those people with SCI and neurogenic bladder. Non-antibiotic prevention is needed to prevent UTI without increasing the antimicrobial resistance burden [5].

Probiotics are defined as a preparation containing viable, defined micro-organisms in sufficient numbers, which alter the microflora [by implantation or colonization] in a compartment of the host and thus exert beneficial health effects in this host [6]. Reid postulated that probiotics could reduce antibiotic related superinfections, disrupt bacterial biofilms, and enhance generalised mucosal immunity in the gastrointestinal and genitourinary systems [7]. In a systematic review conducted by Falagas et al., it was concluded that several probiotics tested, e.g. Lactobacillus rhamnosus GR-1 and Lactobacillus fermentum RC-14, delivered either intravaginally or orally, were efficacious in restoring vaginal flora and in preventing recurrent UTIs in women [8]. In another trial, Manley et al. demonstrated clearance of vancomycin-related enterococci in stool after treatment with Lactobacillus rhamnosus GG [9].

There are currently no known trials of oral probiotics and its efficacy in prevention of UTIs in people with neurogenic bladders. Darouiche and others have conducted more invasive trials involving inoculating neurogenic bladders with benign strains of Escherichia coli and showed this approach was effective at lowering the rate of UTIs per year [10–12].

### Study aims

#### Primary aim

To test the effectiveness of combination oral probiotic therapy Lactobacillus reuteri RC-14 + Lactobacillus rhamnosus GR-1 [RC14-GR1 capsules] and/or Lactobacillus rhamnosus GG + Bifidobacterium BB-12 [LGG-BB12 capsules] in preventing UTI in people with SCI compared to placebo.

#### Secondary aims

- To examine whether probiotics may change or prevent colonisation or infection with MROs in persons with SCI.
- To examine the effects of probiotics on bowel function in persons with SCI
- Examination of indwelling and suprapubic catheters to determine:
- How probiotic intervention affects microbial community composition in the urine and urinary catheter.
- Differences between microbial communities in individuals who are symptomatic versus asymptomatic for UTI.
- To estimate the cost-effectiveness of probiotics in persons with SCI

A randomised controlled trial [RCT] was selected as the design most likely to provide a definitive conclusion to the primary aim.

## Methods/design

This is a prospective multi-site randomised, double-blind, double-dummy, placebo-controlled factorial design trial conducted in the state of New South Wales [NSW] Australia, in order to test the effectiveness of two probiotic therapies in preventing UTI in people with SCI. Participants will be recruited from the NSW SCI community and all the specialist SCI units in NSW hospitals, including their regional and rural affiliations. These units are located at the Prince of Wales Hospital [POWH], Royal Rehabilitation Centre Sydney [RRCS] and Royal North Shore Hospital [RNSH].

### Ethics approval

A multi-site ethics approval was obtained from the Human Research Ethics Committee [HREC] at each of the three SCI units in NSW, Australia. HREC Protocol no: 1008-282-CTN-GG [POWH SSA 1008-282 CTN, RR SSA 11/SSA03, RNSH SSA10/HAWKE/171].

The protocol for catheter sampling and culture independent technique of bacterial community identification was categorised as a low-risk study with separate ethics approval obtained from the HREC at each site [POWH HREC ref no. 11/036, RNSH HREC/10/HARBR/102 and SSA/10/HAWKE/171].

The trial was registered with the Australian New Zealand Clinical Trials Registry [ACTRN 12610000512022] on 21 June 2010. Informed consent will be provided prior to recruitment and participation. Participant recruitment commenced in April 2011.

### Sample size

The trial uses a factorial design which allows the two probiotics to be compared with placebo simultaneously without inflating the sample size, on the assumption that they do not interact with each other. [Refer Table 1].

**Table 1:** Study design for ProSCIUTTU

	LGG -BB12 (B) Active (186)	LGG -BB12 (b) Placebo (186)
GR1-RC14 (A) Active (186)	AB or Intervention A (93)	Ab or Intervention B (93)
GR1-RC14 (a) Placebo (186)	aB or Intervention C (93)	ab or Intervention D (93)

UTI prevention: In our previous RCT with the same study population [4], 45 % of participants had a symptomatic UTI within six months. To have 80 % power to detect [at 5 % two-sided significance level] a 30 % reduction in the treatment group requires a total sample size of 350. Allowing for a 5 % loss to follow-up a final sample size of 372 is required, 93 participants being randomly allocated to each of the four study groups.

MRO treatment: It is expected that approximately 40 % participants will be MRO-positive at enrolment. Assuming 5-10 % become MRO-negative in the control group, a 15–20 % reduction in MRO-positive colonisation rate with probiotics would be detectable as significant at the 5 % level, with 80 % power, with a sample size of 372.

### Randomisation and blinding of assessors

A simple stratified [computer generated] randomisation protocol is used. JS is responsible for generating the allocation sequence. Randomisation is stratified by bladder management types [indwelling/suprapubic vs intermittent catheters vs condom drainage/reflex voiding] as well as inpatient/outpatient status. Randomisation occurs following participant’s compliance check at Day 4. One central pharmacy is responsible for the assignment and distribution of the intervention for the entire study. All four treatment regimens will be indistinguishable by appearance and taste, and all participants receive the same quantity of tablets. All clinical staff, researchers and participants remain blind to treatment allocation throughout this process. An audit of randomisation, product allocation and dispensing stock will be performed at the completion of the study by MT, who is not affiliated with the final analysis and the clinical management of the study or study participants.

### Participants

All participants are to be over 18 years of age and are required to provide written consent. All participants with known neurogenic bladder as a result of SCI who meet inclusion criteria and gave written consent are enrolled. BL, ST, SR, JK, LB, GW and CBR are responsible for enrolling participants.

Inclusion criteria:

1. Had a known neurogenic bladder;
2. Had a stable SCI or stable multiple sclerosis with a known spinal demyelinating lesion;
3. Had a stable bladder management technique [i.e. not receiving bladder management education for at least 4 weeks] and using a bladder management technique such as indwelling catheter, suprapubic catheter, clean intermittent self-catheterisation or reflex/condom drainage;
4. Agreed to fortnightly telephone consultation for themselves and their care team during the six month study period;
5. Agreed not to take any other probiotic in addition to the allocated intervention during the course of the study. This includes all oral or topical preparations of yoghurt and urinary antiseptics [e.g. methenamine hippurate (hiprex) or cranberry preparations].

Exclusion criteria:

1. Receiving bladder management education within the last 4 weeks;
2. Being treated for, or symptomatic from a current infection or long-standing pressure sore;
3. Known to have a complex bladder disturbance requiring surgical intervention e.g. known cystoplasty, renal or bladder calculus, significant hydronephrosis, or current pyelonephritis;
4. Known to have chronic open wound/s or known long-standing osteomyelitis [greater than 6 weeks];
5. On long-term antibiotic therapy for any indication;
6. Known to have a history of adverse drug reaction to yoghurt products or a demonstrated intolerance to the probiotics used. Lactose intolerance was NOT an exclusion criterion;
7. Known to have severe renal or hepatic failure;
8. Requiring full [invasive] mechanical ventilation;
9. Receiving immunosuppressant medications or have an underlying immunosuppressive disease [for example HIV or end-stage/ progressive diabetes mellitus, multiple sclerosis or cerebrovascular disease];
10. Planning to have oral surgery during the intervention period;
11. Concurrently enrolled in another intervention study [observational studies or inclusion following completion of another study was allowed].

**Fig 1:**
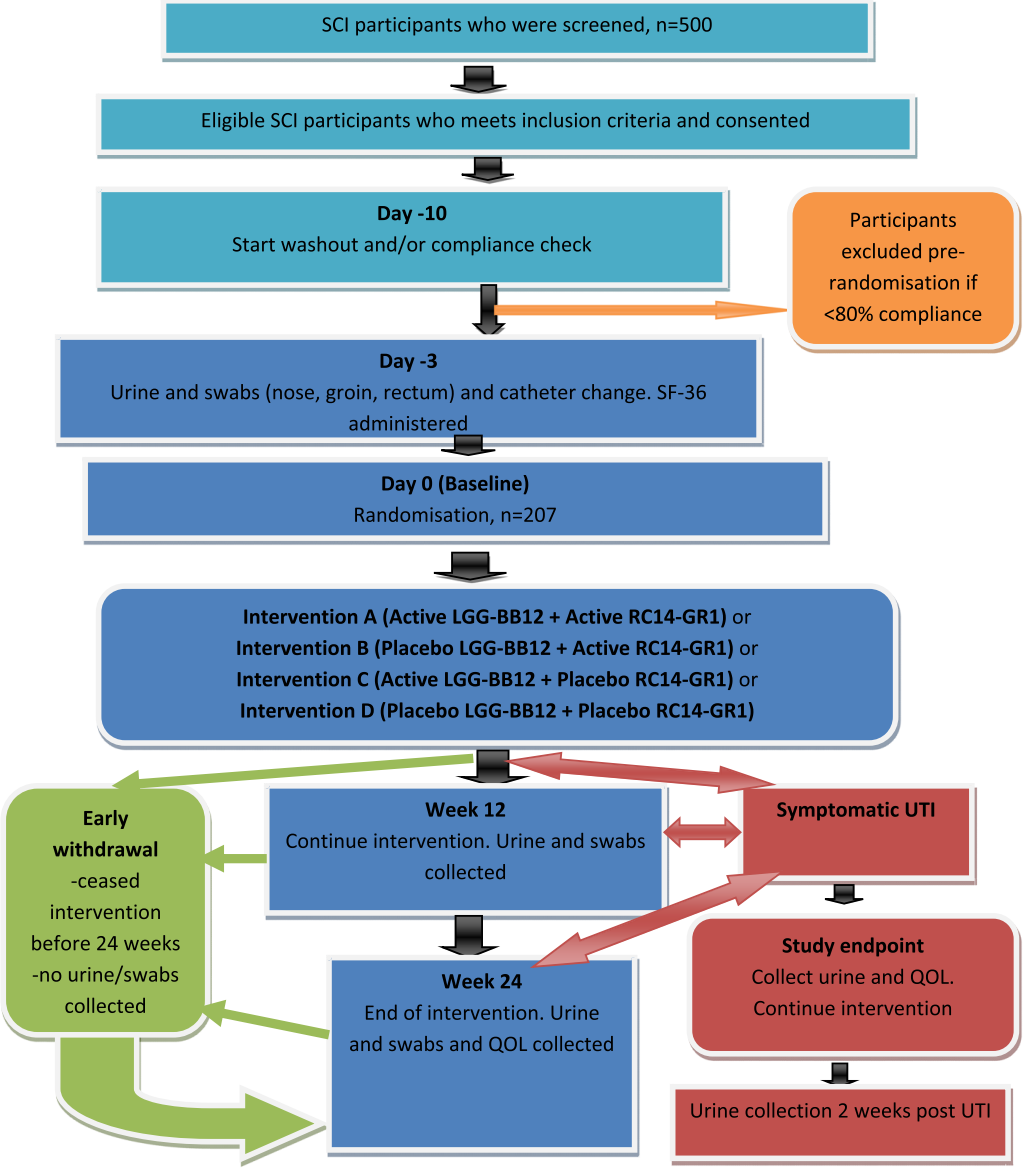
Participant Study Flow Chart for ProSCIUTTU

Each participant is enrolled for a six month study period, which includes 24 weeks of treatment [see Fig. 1]. Each participant randomised is required to take two tablets orally each day consisting of either RC14-GR1 + LGG-BB12 or RC14-GR1 + placebo or LGG-BB12 + placebo or 2 placebo tablets.

Active Interventions:

1. GR1-RC14. Concentration per capsule is 5.4 × 10^9^ colony forming units.
2. LGG-BB12. Concentration per capsule is 7 × 10^9^ colony forming units.

Participants will be assessed at Day 0, 3 months and 6 months, supported by fortnightly phone calls to determine health status and confirm intercurrent symptomatic UTI status. [Fig. 1] Following witnessed informed consent, evaluations conducted will be:

- Intervention issues and compliance.
- Quality of life assessment with the Short Form Health Survey [SF-36] – baseline and 6 months plus study endpoint if reached.
- Microbiological swabs of rectum, nose and groin, urine culture and collection of urinary catheters for participants with indwelling or suprapubic catheters – baseline, 3 months and 6 months. Urine cultures also performed if at study endpoint. Specific instructions for sampling were given by study co-ordinator to research assistants and community nurses performing the swabs to ensure consistency.
- Bowel questionnaire [13, 14] - baseline and 3 months.

### Catheter-bioflora analysis

The indwelling urethral and suprapubic catheter biofilm is examined as a proxy for the urinary tract microbial community. Culture-independent techniques in profiling human microbes will be used to determine the composition of adherent microbes through the examination of the bacterial 16S rDNA gene by Terminal Restriction Fragment Length Polymorphism [TRFLP] [15] and via next-generation sequencing [16–19]

Samples are selected irrespective of interventional grouping.

Samples are also selected from groups with recurrent symptomatic UTI compared to no-UTI symptoms over the study follow-up period. All TRFLP and sequencing analysis will be conducted blinded by the use of a participant identification key that de-identifies the data.

TRFLP is done in collaboration with the Ramaciotti Centre for Genomics, University of New South Wales and sequencing through the Singapore Centre for Environmental Life Sciences Engineering at Nanyang Technological University, Singapore.

### Study endpoints

**Fig 2:**
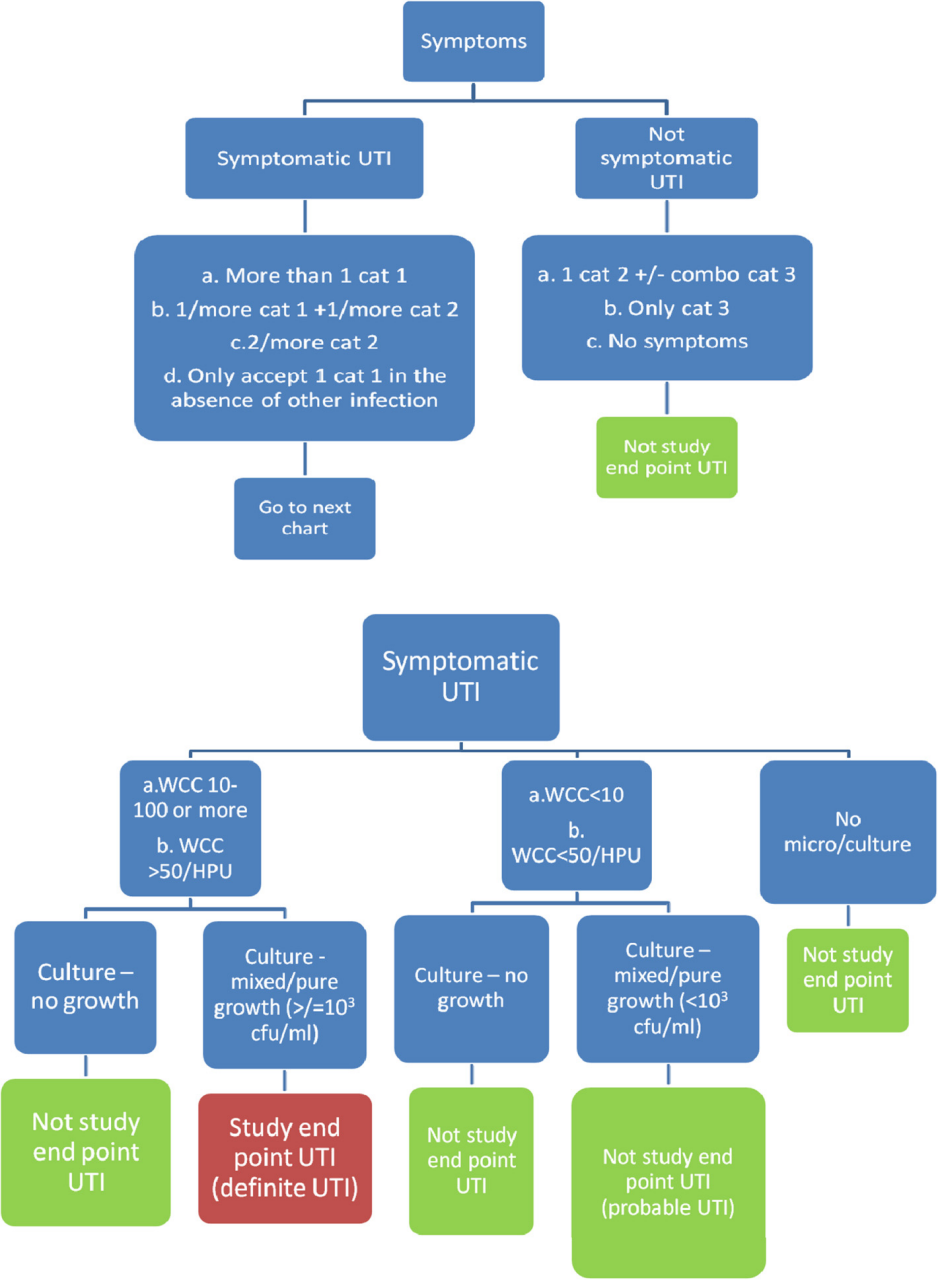
Definition of primary endpoint UTI for ProSCIUTTU (need to refer to Table 2)

1. The primary outcome measure is the time from randomisation to occurrence of “symptomatic UTI” [Fig. 2]. The date of the endpoint is the date participants develop symptoms consistent with a “symptomatic UTI” as per the algorithm, not the date participants start developing any symptoms. Table 2 outlines the definition of “symptomatic UTI” as primary endpoint for ProSCIUTTU. For participants who do not experience a “symptomatic UTI”, the primary outcome is at six months. Participants who cease intervention early are followed up until the end of the study period.

**Table 2:** Definition of "symptomatic UTI" as primary endpoint for ProSCIUTTU (need to refer to Fig. 2). Use the following table to assess “Category 1”, or Two “Category 2” and any “Category 3” Symptoms: All symptoms should be asked in each category

“*Category 1*” Symptoms:*	“Category 2” Symptoms:*	“Category 3” Symptoms:
	Two or more	In themselves not enough to lead to treatment but recorded for International Spinal Cord Injury Urinary Tract Infection Datasets Compatibility
• Temperature: Greater than 38 ° C core Greater than 37.5 ° C per axilla • New or increasing symptoms of Autonomic Dysreflexia, as detected by any of the following signs: *Pulse < 50 or increased flushing or sweating or headache AND increased B.P Diastolic or Systolic > 25 % usual baseline.*	• Increased Frequency of Muscle Spasms or spasticity • Failure of usual control of urinary incontinence- *any of the following constitutes fulfillment of this category* -Bladder Spasm -Urinary frequency or need for increased catheterization -Urinary Retention -Urinary Urgency -Leaking around catheter site or per urethra if have suprapubic catheter • New Scrotal/Loin/Abdominal Discomfort unexplained by other pathology - *any of the following constitutes fulfillment of this category* -Abdominal Pain -Bladder/Suprapubic Pain -Loin/Back Pain -Scrotal Pain -Dysuria	*any of the following constitutes fulfillment of this category* -Anxiety/uneasiness -Feeling tired -Feeling sick -Arthralgias/Body Aches -Chills -Diaphoresis/sweating -Cloudy Urine -Foul smelling urine -Blood in urine *haematuria* -Catheter blockage

*Content adapted and modified from Box 1 of Spinal-injured neuropathic bladder antisepsis (SINBA) trial [4]
2. The secondary endpoint is time to change of MRO colonisation status as determined by two successive cultures [See guidelines for MRO change or clearance in Additional file 1].

### Data analysis

All analyses of outcomes will be by intention to treat, apart from safety outcomes which will be according to actual treatment received. Primary and MRO outcomes will be analysed using survival analysis. Cox regression modelling will be performed to test the effect of each treatment while allowing for the other, assuming no interaction effect. Hazard ratios and Kaplan-Meier survival curves will be used to summarise results. The extremely high prevalence of MRO in SCI will also allow us to explore whether probiotics can treat [or prevent] MRO colonisation in this group.

A survey was sent out to selected co-authors for determining the strength of association of several variables in regards to UTI in the SCI population [pre hoc review]. Only variables which have strong or moderate association will be included in the analysis.

Biofilms will be analysed using a combination of RNA based meta-community sequencing, TRFLP fingerprinting and culture based methods.

### Trial data management

The data will be collected on trial specific case record forms. OM is responsible for designing and maintaining the trial database. Following each study visit, a study team member will ensure data is complete. Databases will be commissioned within the SCI units and will contain non-identifiable data. Re-identifiable data will be available for use only by the study team. Primary outcome measure endpoint determination will be verified by BL and ST. The two assessors will be blinded by each other’s assessment. Discrepancies will be decided by a third investigator [KC].

### Feasability, safety, efficacy

#### Efficacy

The primary study endpoint is symptomatic UTI with microbiological evidence of infection [refer to Fig. 2 and Table 2]. However, other secondary measures of interest include:

- Clinical infection.
- Hospital admissions and intensive care unit admissions related to infection.
- A diagnosis of laboratory infection defined by a positive blood culture.
- Clinical adverse events [grade 3–4] regardless of causality.
- All causes of mortality.
- Use of antibiotics.
- Change in of MRO colonisation/infection status as defined by two consecutive MRO swabs three months apart.
- Modifications of bladder management.
- SF-36
- A cost-effectiveness analysis will be undertaken using SF-6D utility weights derived from the SF-36. In addition to antibiotic use, the following resource data will also be collected during the study:

- Use of isolation precautions: Single room; Personal Protective Equipment [PPE];
- Isolation ward;
- Terminal clean
- Infection control auditing

### Safety monitoring

An independent Safety Monitoring Committee [SMC] is established. Clinicians or investigators responsible for the clinical care of study participants were not permitted to be members of the SMC. The SMC will monitor the trial and review safety data by treatment allocation. Safety monitoring will be carried out at various intervals through the trial depending on frequency of adverse events. The Committee will review laboratory data, Grade 3, Grade 4 and Grade 5 adverse events and serious adverse events [SAEs] and adverse events leading to cessation of study therapy [refer Table 3]. A summary of safety data will be undertaken when all recruited participants have completed 20 weeks on study.

**Table 3:** Severity grade of adverse events

ESTIMATING SEVERITY GRADE				
PARAMETER	GRADE 1	GRADE 2	GRADE 3	GRADE 4
Clinical adverse event	Symptoms causing no or minimal interference with usual social & functional activities	Symptoms causing greater than minimal interference with usual social & functional activities	Symptoms causing inability to perform usual social & functional activities	Symptoms causing inability to perform basic self-care functions OR Medical or operative intervention indicated to prevent permanent impairment, persistent disability

Grades 1 and 2 Laboratory Abnormality or Clinical Event Continue intervention at the discretion of the investigator

Grade 3 Laboratory Abnormality or Clinical Event Grade 3 clinically significant laboratory abnormalities should be confirmed by repeat testing within three to five calendar days of receipt of results and before discontinuation, unless such a delay is not consistent with good medical practice For grade 3 clinical events, continue if the event is considered to be unrelated to the intervention. For a grade 3 clinical event, or clinically significant laboratory abnormality confirmed by repeat testing, that is considered to be related to the intervention, both oral and bodywash interventions should be withheld until the toxicity returns to ≤ grade 2. When restarting following resolution of the adverse event, both interventions to be restarted simultaneously following discussion with the study monitor

Grade 4 Laboratory Abnormality or Clinical Event For grade 4 clinical event or clinically significant laboratory abnormality confirmed by repeat testing that is considered related to the intervention, the intervention should be permanently discontinued and subjects managed according to local practice. The subject should be followed as clinically indicated until the event resolves to baseline, or is otherwise explained, whichever occurs first. Study interventions may be continued without modification for non-clinically significant grade 4 laboratory abnormality (e.g. triglyceride elevation that is non-fasting or that can be medically managed) or clinical event considered unrelated to the study intervention

The SMC Chairman had no formal affiliation with the trial and coordinated this process.

### Project governance and administration support

The chief investigator Dr. Bon San Bonne Lee will be responsible for overall project management, but is assisted and advised by a project steering committee comprised of the collaborating researchers and administrative support from the administering institution [NeuRA]. The project steering committee will meet regularly and all agendas and minutes circulated to all stakeholders.

### Trial status

Trial commencement date: April 2011

Trial follow-up completion date: March 2014

Number of participants recruited: 207

## Additional file

Additional file 1: Guidelines for MRO change and clearance. (DOC 27 kb)

## Abbreviations

BB12: bifidobacterium BB-12
GR1: lactobacillus rhamnosus GR-1
LGG: lactobacillus rhamnosus GG
MRO: multi-resistant organism
MS: multiple sclerosis
NHMRC: National Health and Medical Research Council of Australia
PPE: personal protective equipment
RC14: lactobacillus reuteri RC-14
RNA: ribonucleic acid
SAE: serious adverse event
SF-36: short form health survey
SCI: spinal cord injury
SMC: safety monitoring committee
TRFLP: terminal restriction fragment length polymorphism
UTI: urinary tract infection

## References

[1] Waites KB, Y-y C, DeVivo MJ, Canupp KC, Moser SA (2000). Antimicrobial resistance in gram-negative bacteria isolated from the urinary tract in community-residing persons with spinal cord injury. Arch Phys Med Rehabil.

[2] Mylotte JM, Kahler L, Graham R, Young L, Goodnough S (2000). Prospective surveillance for antibiotic-resistant organisms in patients with spinal cord injury admitted to an acute rehabilitation unit. Am J Infect Control.

[3] Pittet D (2005). Infection control and quality health care in the new millenium. Am J Infect Control.

[4] Lee B, Haran M, Hunt L, Simpson J, Marial O, Rutkowski S (2007). Spinal-injured neuropathic bladder antisepsis (SINBA) trial. Spinal Cord.

[5] Murphy DP, Lampert V (2003). Current implications of drug resistance in spinal cord injury. Am J Phys Med Rehabil.

[6] Schrezenmeir J, de Vrese M (2001). Probiotics, prebiotics, and synbiotics—approaching a definition. Am J Clin Nutr.

[7] Reid G (2006). Probiotics to prevent the need for, and augment the use of, antibiotics. Can J Infect Dis Med Microbiol.

[8] Falagas ME, Betsi GI, Tokas T, Athanasiou S (2006). Probiotics for prevention of recurrent urinary tract infections in women. Drugs.

[9] Manley KJFM, Maynall BC, Power DA (2007). Probiotic treatment of vancomycin-resistant enterococci : a randomised controlled trial. Med J Australia.

[10] Darouiche RO, Donovan WH, Del Terzo M, Thornby JI, Rudy DC, Hull RA (2001). Pilot trial of bacterial interference for preventing urinary tract infection. Urology.

[11] Darouiche RO, Thornby JI, Stewart CC, Donovan WH, Hull RA (2005). Bacterial interference for prevention of urinary tract infection: A prospective, randomized, placebo-controlled, double-blind pilot trial. Clin Infect Dis.

[12] Prasad A, Cevallos ME, Riosa S, Darouiche RO, Trautner BW (2009). A bacterial interference strategy for prevention of UTI in persons practicing intermittent catheterization. Spinal Cord.

[13] Krogh K, Perkash I, Stiens S, Biering-Sørensen F (2008). International bowel function basic spinal cord injury data set. Spinal Cord.

[14] Juul T, Bazzocchi G, Coggrave M, Johannesen I, Hansen R, Thiyagarajan C (2011). Reliability of the international spinal cord injury bowel function basic and extended data sets. Spinal Cord.

[15] Liu W-T, Marsh TL, Cheng H, Forney LJ (1997). Characterization of microbial diversity by determining terminal restriction fragment length polymorphisms of genes encoding 16S rRNA. Appl Environ Microbiol.

[16] Coolen MJ, Post E, Davis CC, Forney LJ (2005). Characterization of microbial communities found in the human vagina by analysis of terminal restriction fragment length polymorphisms of 16S rRNA genes. Appl Environ Microbiol.

[17] Li F, Hullar MA, Lampe JW (2007). Optimization of terminal restriction fragment polymorphism (TRFLP) analysis of human gut microbiota. J Microbiol Methods.

[18] Khoruts A, Dicksved J, Jansson JK, Sadowsky MJ (2010). Changes in the composition of the human fecal microbiome after bacteriotherapy for recurrent Clostridium difficile-associated diarrhea. J Clin Gastroenterol.

[19] Fouts DE, Pieper R, Szpakowski S, Pohl H, Knoblach S, Suh M-J (2012). Integrated next-generation sequencing of 16S rDNA and metaproteomics differentiate the healthy urine microbiome from asymptomatic bacteriuria in neuropathic bladder associated with spinal cord injury. J Transl Med.

