# Supplementary material for: Probiotics [LGG-BB12 or RC14-GR1] versus placebo as prophylaxis for urinary tract infection in persons with spinal cord injury [ProSCIUTTU]: a study protocol for a randomised controlled trial
Source: BMC Urol. 2016 Apr 16;16:18. doi: 10.1186/s12894-016-0136-8 (PMC4833921; doi:10.1186/s12894-016-0136-8)
Supplement: Additional file 1: — Guidelines for MRO change and clearance. (DOC 27 kb) [file 12894_2016_136_MOESM1_ESM.doc]

**Supplementary material for ProSCIUTTU**

**GUIDELINES FOR MRO CHANGE and CLEARANCE**

All the following criteria should be satisfied prior to certifying that a patient has cleared a particular MRO:

**CATEGORY 1 -MRO change (primary endpoint)**

- No exposure to systemic or topical antibiotics, urinary antiseptics or antiseptic body wash** for at least 2 weeks prior to screening;
- MRO status change within the first 3 months of follow up.
- If antiseptic body washes have been used for greater than 3 months they will not be ceased

Any change in MRO status after 3 months will be a secondary endpoint

**CATEGORY 2 - MRO CLEARANCE Sustained disease survival**

**•** MRO status change within the first 3 months

**• Two** consecutive negative screens at least 2 weeks apart

**• AND** a confirmed persistence of MRO status change at study EOT(month 6 or month 7).

**All topical antiseptic agents that a patient is using, including commencement and ceasing dates will be recorded. Any agents commenced within 1 month at baseline will be assessed and either continued or ceased during the washout period.

**DEFINITIONS OF CLINICAL SYMPTOMS**

**Abdominal pain**: patient complains of pain that may be generalized or localized from below the chest to above the groin. It may be sharp, dull, cramping or colicky (sudden starting and stopping)

**Anxiety/uneasiness:** patient complains of a vague uneasy feeling, the source of which is often non specific or unknown to the individual.

**Arthralgia/body aches**: patient complains of pain in one or more joints/pain throughout the body

**Autonomic dysreflexia:** In SCI individuals with injuries at T6 and above –patient complains of the feeling of a sudden onset of elevated blood pressure, and other symptoms such as headache, sweating, flushing brought on by a noxious stimuli such as bladder distension/ bladder infection). Refer to PVA Clinical Practice Guideline.

**Back pain:** Pain in the lower back below the rib cage – complaints of pain located on one or other side of the back just below the ribs

**Bladder pain:** complaints of pain felt suprapubically or retropubically. Usually increases with bladder filling, and may persist after voiding

**Bladder spasms:** feeling of the bladder squeezing usually due to bladder contractions (over active bladder). It may cause urinary incontinence or leaking around a catheter.

**Blood in the urine:** Visible blood with or without clots in urine

**Change in bowel habits:** complaints of a new onset of constipation or soft or loose stools/diarrhoea

**Chills:** a sensation of cold often accompanied by shivering

**Cloudy urine:** complaints that the urine is not clear. There may complaints of mucus or sediment.

**Dysuria:** pain and discomfort when voiding. Usually associated with localized inflammation but may be referred pain from the bladder, prostate or sphincter.

**Fatigue, sleepiness, tiredness:** complaints of generalized weariness

**Fever:** feeling that body temperature is above normal.

**Headache:** pain in the head (cephalalgia) that may be associated with autonomic dysreflexia

**Nausea/emesis:** stomach discomfort with a feeling to vomit or actual vomiting

**Neuropathic pain:** abnormal, often painful sensation in a part of the body due to abnormal nerve function

**Scrotal pain:** complaints of pain, which may or may not be localized to the testis, epididymis, cord structures or scrotal skin.

**Spasticity:** complaints of new or increased muscular hypertonicity with increased resistance to stretch

**Urinary frequency (or need for increased catheterizations):** the complaint from the patient that considers he/she voids too often.

**Urinary incontinence:** failure of control or leaking around the catheter: The complaint of any involuntary leakage of urine. This may of may not be associated with urgency.

**Urinary retention:** feeling of bladder distension or incomplete bladder emptying due to an inability to void. This may be due to an obstruction to the bladder outlet, sphincter spasticity or a bladder that does not contract

**Urinary urgency:** The complaint of a sudden compelling desire to pass urine, which is difficult to defer.

* Some content adapted and modified from the 1992 National Institute on Disability and Rehabilitation Research Statement on symptomatic urinary tract infections in the spinal cord injured; Dr B Lee, Dr G Kotsiou (RNSH Microbiology Department)
